# Extension of the DNAJB2a isoform in a dominant neuromyopathy family

**DOI:** 10.1093/hmg/ddad058

**Published:** 2023-04-18

**Authors:** Jaakko Sarparanta, Per Harald Jonson, Jens Reimann, Anna Vihola, Helena Luque, Sini Penttilä, Mridul Johari, Marco Savarese, Peter Hackman, Cornelia Kornblum, Bjarne Udd

**Affiliations:** Folkhälsan Research Center, Helsinki, Finland and Medicum, University of Helsinki, FI-00290 Helsinki, Finland; Folkhälsan Research Center, Helsinki, Finland and Medicum, University of Helsinki, FI-00290 Helsinki, Finland; Klinik und Poliklinik für Neurologie, Sektion Neuromuskuläre Erkrankungen, Universitätsklinikum Bonn, D-53127 Bonn, Germany; Folkhälsan Research Center, Helsinki, Finland and Medicum, University of Helsinki, FI-00290 Helsinki, Finland; Neuromuscular Research Center, Tampere University Hospital and Fimlab Laboratories, FI-33520 Tampere, Finland; Folkhälsan Research Center, Helsinki, Finland and Medicum, University of Helsinki, FI-00290 Helsinki, Finland; Neuromuscular Research Center, Tampere University Hospital and Fimlab Laboratories, FI-33520 Tampere, Finland; Folkhälsan Research Center, Helsinki, Finland and Medicum, University of Helsinki, FI-00290 Helsinki, Finland; Harry Perkins Institute of Medical Research, Centre for Medical Research, University of Western Australia, Nedlands WA, Australia; Folkhälsan Research Center, Helsinki, Finland and Medicum, University of Helsinki, FI-00290 Helsinki, Finland; Folkhälsan Research Center, Helsinki, Finland and Medicum, University of Helsinki, FI-00290 Helsinki, Finland; Klinik und Poliklinik für Neurologie, Sektion Neuromuskuläre Erkrankungen, Universitätsklinikum Bonn, D-53127 Bonn, Germany; Folkhälsan Research Center, Helsinki, Finland and Medicum, University of Helsinki, FI-00290 Helsinki, Finland; Neuromuscular Research Center, Tampere University Hospital and Fimlab Laboratories, FI-33520 Tampere, Finland

## Abstract

Recessive mutations in the *DNAJB2* gene, encoding the J-domain co-chaperones DNAJB2a and DNAJB2b, have previously been reported as the genetic cause of progressive peripheral neuropathies, rarely involving pyramidal signs, parkinsonism and myopathy. We describe here a family with the first dominantly acting *DNAJB2* mutation resulting in a late-onset neuromyopathy phenotype. The c.832 T > G p.(^*^278Glyext^*^83) mutation abolishes the stop codon of the DNAJB2a isoform resulting in a C-terminal extension of the protein, with no direct effect predicted on the DNAJB2b isoform of the protein. Analysis of the muscle biopsy showed reduction of both protein isoforms. In functional studies, the mutant protein mislocalized to the endoplasmic reticulum due to a transmembrane helix in the C-terminal extension. The mutant protein underwent rapid proteasomal degradation and also increased the turnover of co-expressed wild-type DNAJB2a, potentially explaining the reduced protein amount in the patient muscle tissue. In line with this dominant negative effect, both wild-type and mutant DNAJB2a were shown to form polydisperse oligomers.

## Introduction

The J-domain proteins (JDPs, also known as DNAJ proteins, or Hsp40) are a large and diverse group of cochaperones essential for cellular protein quality control (PQC). The defining feature of all JDPs is the J domain, which stimulates the ATPase activity of HSPA (Hsp70) chaperones, enabling the chaperone cycle, i.e. alternating binding and release of client (substrate) proteins. JDPs may also recognize and present clients to the HSPA machinery and affect their triage to refolding and/or degradative pathways. Some JDPs also possess HSPA-independent anti-aggregation activity (1,2).

The *DNAJB2* (HSJ1) gene encodes two JDP cochaperones with alternative C-terminal parts generated through an alternative splice site within the last exon (3). The shorter DNAJB2a (HSJ1a) isoform is localized to the nucleus and cytoplasm, while the longer DNAJB2b (HSJ1b) isoform has a geranylgeranyl anchor attaching it to the cytoplasmic face of the endoplasmic reticulum (4). Common to both isoforms are the N-terminal J domain (JD), which is followed by glycine/phenylalanine-rich (G/F) domain and a C-terminal domain (CTD) containing a short serine-rich (SR) region and two ubiquitin-interacting motifs (UIMs) (4,5). DNAJB2 shows highest expression in neurons, particularly in the neocortex, and lower levels have been reported in various cells and tissues (3,4,6). In most tissues, DNAJB2b is the predominant isoform (4,6–8). A notable exception is human skeletal muscle, where the levels of the protein isoforms have been reported to be roughly equal (6).

The principal function of DNAJB2 is to mediate degradation of HSPA client proteins through the ubiquitin–proteasome system (UPS). DNAJB2 interacts with polyubiquitinated proteins and the proteasome through its UIMs, and cooperates with STUB1 (CHIP) to promote client ubiquitination and sorting to UPS (5,9). The membrane-anchored DNAJB2b isoform has been shown to promote the proteasomal degradation of endoplasmic reticulum (ER)-located proteins via the ER-associated degradation pathway (5). Studies using a variety of model systems and clients have demonstrated that DNAJB2 also has intrinsic chaperone activity and PQC functions independent of the HSPA system and/or UIMs (reviewed in Sarparanta *et al*. 10). DNAJB2a has, for instance, been shown to efficiently counteract TARDBP (TDP-43) aggregation by promoting its HSPA-mediated refolding (11). For a more detailed discussion on the reported functions of DNAJB2, we refer to our recent review (10).

Recessive mutations in *DNAJB2* cause a spectrum of progressive peripheral axonal neuropathies, ranging from a pure motor phenotype [distal hereditary motor neuropathy (dHMN), a.k.a. distal spinal muscular atrophy] to sensorimotor axonal Charcot–Marie–Tooth disease (CMT2) (7,8,12–21). Pyramidal tract signs and Parkinson’s disease (PD) have been described in some patients, and hearing loss in one family (14–17,21). Recently, rimmed-vacuolar myopathy in combination with dHMN was described in a patient homozygous for a *DNAJB2* missense mutation (20). The clinical course of DNAJB2-related neuropathies is typically moderate to severe, with onset in the second to third decades and progressing to severe muscle weakness over the course of the disease (7,8,13,15–21).

All the hitherto described *DNAJB2* mutations (Table 1) are recessive and apparently act through a loss-of-function mechanism. Most of the mutations are demonstrated or expected to completely abolish DNAJB2 expression (7,8,21). In addition, a few missense mutations have been described, most of them affecting the J domain (8,12,17,20). On the basis of their recessive mode of action and their clinical consequences being comparable with the null alleles, the missense changes are likely to act by inactivating or destabilizing the protein. The downstream mechanisms leading from DNAJB2 loss of function to neuropathy are largely unknown, but accumulation of phosphorylated TARDBP in patient skin biopsies suggests that TARDBP aggregation has a potential role in the pathomechanism (21). Moreover, axonal accumulation of phosphorylated α-synuclein was specifically observed in a skin biopsy from a patient with PD-associated CMT2 due to a *DNAJB2* mutation (21).

**Table 1:** Published DNAJB2 mutations

DNA change	Effect	Clinical phenotype	Ref.
c.14A > G	p.Tyr5Cys	CMT2	(8,12)
c.125C > A	p.Ala42Asp	dHMN, YOPD, pyramidal	(17)
c.145delG	p.Val49Trpfs^*^25	CTM2, PD, hearing loss	(21)
c.176-3C > G	Splice change	dHMN, pyramidal	(16)
c.184C > T	p.Arg62Trp	dHMN, myopathy	(20)
c.229 + 1G > A	Splice change	dHMN	(8,18)
c.310delC	p.R104Gfs^*^97	CMT2	(13)
c.352 + 1G > A	Splice change	dHMN, YOPD	(7,14,17)
c.620-1G > A^a^	Splice change	CMT2	(13)
c.700A > T^b^	p.Thr234Ser	dHMN	(19)
g.219277938_219281781del	Likely null	dHMN, YOPD	(15)

Reference sequences: NM_001039550.1 (cDNA), NP_001034639.1 (protein), NC_000002.12 (genomic)

dHMN: distal hereditary motor neuropathy; CMT2: axonal Charcot–Marie–Tooth disease; (YO)PD: (young onset) Parkinson’s disease

^a^Annotated as c.619-1G > A in the original publication

^b^Classified as a variant of unknown significance (VUS) in the original publication

## Results

### Patients

**Figure 1:**
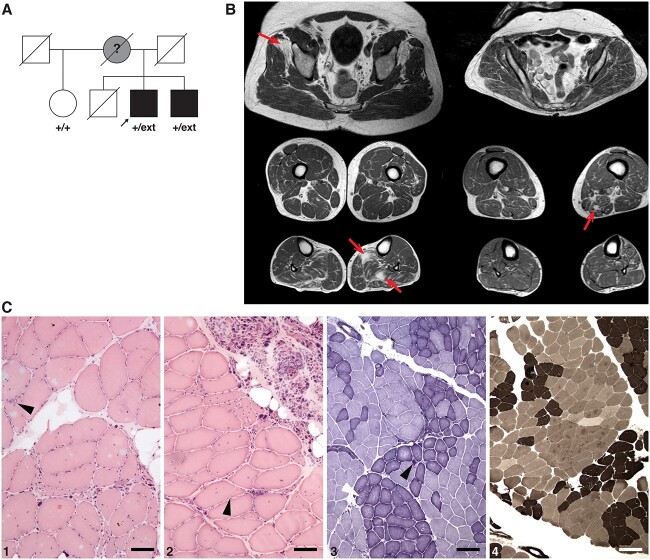
Patients and clinical findings. (**A**) Pedigree of our family. The *DNAJB2* genotype (+, wild-type; ext, c.832 T > G p.^*^278Glyext^*^83) is shown for the family members available for genetic analysis. (**B**) Muscle MRIs of the proband (left) show diffuse neurogenic degenerative change in the soleus and gastrocnemius lateralis muscles of the lower legs but also myopathic-dystrophic fatty replacement focal changes in anterior parts of gluteus minimus and left soleus (arrows). Degenerative changes in the younger brother (right) are similar but milder with more neurogenic but also spots of myopathic replacement in the left adductor magnus (arrow) and the outer part of both peroneus longus muscles. (**C**) Gastrocnemius muscle biopsy of the proband (1 and 2 haematoxylin/eosin, 3 NADH and 4 ATPase pH 4.6) showing classical neurogenic changes such as fibre type grouping and groups of atrophic fibres together with clear myopathic changes such as rimmed vacuoles (arrowhead in 1), fibre splitting (arrowhead in 2) and heavily increased number of internalized myonuclei. Furthermore, in NADH staining (3), some small dark angulated fibres (black arrowhead in 3) and few moth-eaten fibres (white arrowhead in 3) are found. Scale bars 25 μm for 1 and 2, 100 μm for 3 and 4.

Our proband is a German male first examined at the age of 66 years. He had suffered from balance problems and leg weakness, worsened by exercise, since the fifth decade, walking difficulties since the sixth decade and stocking-like paraesthesias in later years. The proband’s younger brother had no subjective symptoms, but neuromuscular examination revealed muscle hypotrophy of lower legs. A further brother had died in his 60s of cardiac causes without known neurological complaints. Their mother was reported to have suffered from walking difficulties since her early 60s, with a broad-based, slow gait. The proband’s maternal half-sister suffered from an unrelated paternally inherited spinocerebellar ataxia since her 50s (Fig. 1A).

### Clinical and laboratory findings

Neuromuscular examination of the proband at the age of 66 years showed diminished knee reflexes and absent ankle reflexes. There was bilateral foot dorsiflexion weakness Medical Research Council (MRC) grade 4 and plantar flexion grade 3–4. The patient had severe ataxia in tandem gait. No central motor signs, myoclonus or dystonia were present. He reported paraesthesia in the soles of the feet and showed loss of vibration sensation at the ankles, while reduced at the knee level. He reported reduced sense of position of the toes and had mild claw toes. Serum creatine kinase (CK) was in excess of 7× the upper limit of normal (ULN), and laboratory screening revealed increased rheumatoid factor and low vitamin B_12_. Supplementation was started for the latter, and vitamin B_12_ levels normalized over 6 weeks; no macrocytosis or anaemia was observed. His medical history included arterial hypertension and sleep apnoea under CPAP treatment. Chronic renal failure (stage G3a according to Kidney Disease Improving Global Outcomes) due to chronic mesangioproliferative glomerulonephritis type IgA had recently been diagnosed, but creatine values did not worsen over the time of observation, and steroid medication was not used.

Muscle MRI showed both neurogenic and myopathic degenerative changes (Fig. 1B), while head and spinal MRI failed to detect pathology of the brain or spinal cord.

Neurographic examination (Supplementary Material, Table S1) showed clear sensorimotor polyneuropathy with minor signs suggesting demyelination (proximal decreased compound muscle action potential, prolonged F-wave and distal motor latencies), but without slowing of nerve conduction velocities. Transcranial magnetic stimulation (TMS) showed increased total and central motor conduction times to all four limbs. Electromyography (EMG) detected chronic neurogenic changes (increased motor unit potential durations) but no spontaneous activity in upper limb muscles.

Muscle biopsy from the gastrocnemius revealed severe chronic neurogenic changes with signs of extensive reinnervation (broad, double-peaked fibre diameter distribution, fibre type grouping, grouped atrophy) together with myopathic changes (fibre splitting, internalized myonuclei, rimmed vacuoles) (Fig. 1C), whereas biopsy of the sural nerve showed mixed axonal and demyelinizing changes. The highly elevated CK, focal fatty replacement changes on MRI, even in gluteus minimus, and the muscle biopsy findings nevertheless indicate a partly myogenic origin to the disease.

Upon follow-up, the proband’s symptoms and neurophysiological findings had worsened. At the age to 70, examinations showed bilateral foot dorsiflexion weakness MRC grade 3–4 and plantar flexion grade 2, knee flexion and extension now weak at grade 4. There was Trendelenburg gait and mild atrophy of the thighs, in particular the posterior compartment. Atrophy of the anterior and posterior distal leg muscles on the left and normal calf size on the right with few fasciculations were evident. No central motor signs, myoclonus or dystonia were present. He complained of stocking-like hypaesthesia with hyperalgesia of the soles. There was loss of vibration sense at the ankles, and marked claw toes had developed. His serum CK was 2.5× ULN.

Repeat neurophysiological examinations show some progression of the neuropathy, sparing the right peroneal nerve. In TMS, potentials could no longer be recorded from the tibialis anterior muscles. In EMG, all tested limb muscles except right tibialis anterior and paravertebrals showed chronic neurogenic changes, and some spontaneous activity could be detected in the right vastus lateralis and the left tibialis anterior.

The proband’s brother was examined at the age of 66 years showing diminished knee reflexes and absent ankle reflexes. He had mild foot dorsiflexion weakness on the right, grade 4 on the left and weak big toe extension grade 2–3. Anterior and posterior compartments of the legs showed asymmetric (right > left) atrophy. No central motor signs, myoclonus or dystonia were present. He had mild ataxia in tandem gait and reduced vibration sense at the ankles with otherwise normal sensory examination. His serum CK was normal. Muscle MRI of pelvis and lower extremities showed milder neurogenic degenerative changes distally more on the right side (Fig. 1B), and neurography revealed a mild sensorimotor polyneuropathy.

### Molecular genetics

**Figure 2:**
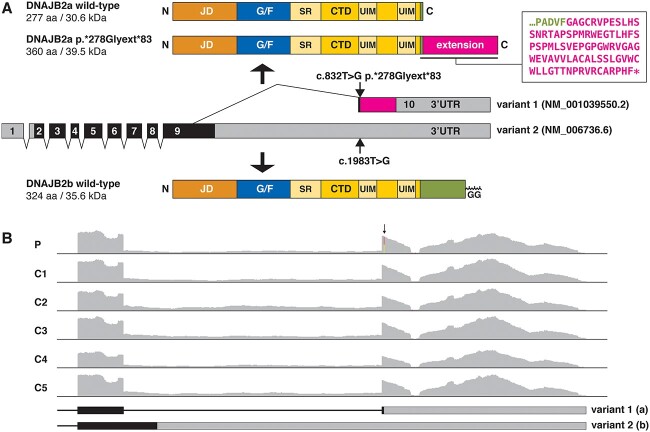
The DNAJB2 mutation. (**A**) The T > G change identified in the proband affects the termination codon of the *DNAJB2* transcript variant 1 (NM_001039550.2) and is predicted to cause a C-terminal extension of the DNAJB2a protein isoform (NP_001034639.1:p.^*^278Glyext^*^83). In variant 2 (NM_006736.6) encoding the DNAJB2b isoform, the altered nucleotide lies within the 3′ UTR and does not affect the protein product. In the diagram of the *DNAJB2* transcript, the non-coding regions are shown in grey, the normal coding regions in black and the extended open reading frame caused by the mutation in magenta. Both the DNAJB2a (top) and DNAJB2b (bottom) proteins contain an N-terminal J domain (JD; orange) followed by a glycine/phenylalanine-rich region (G/F; blue), and a C-terminal domain (CTD; yellow) containing a serine-rich region (SR) and two ubiquitin-interacting motifs (UIMs). The two isoforms differ in their C-terminal parts (green), and DNAJB2b has a C-terminal geranylgeranyl moiety (GG) anchoring it to the endoplasmic reticulum. The extended DNAJB2a protein (p.^*^278Glyext^*^83) produced from the mutant allele has a C-terminal extension of 83 amino acids (magenta; sequence shown in the box). (**B**) RNA sequencing (RNAseq) coverage graphs covering the last exon(s) of the *DNAJB2* transcripts. RNAseq of the proband (P) muscle sample showed equal expression of the wild-type and mutant alleles (arrow) and did not indicate splicing changes or altered isoform ratio compared with other samples run in the same batch (C1–5).

Targeted high-throughput sequencing of the proband with the MyoCap panel identified 20 rare variants (minor allele frequency < 1% in gnomAD_ALL). Of these, the only likely pathogenic variant shared between the proband and his affected brother was the heterozygous stop-loss variant NM_001039550.2:c.832 T > G p.(^*^278Glyext^*^83) in the *DNAJB2* gene. The variant was not previously reported in the gnomAD or ClinVar databases. Sanger sequencing confirmed that the variant was present in the proband and his brother, and absent from their unaffected half-sister (Fig. 1A). The identified variant abolishes the termination codon of the DNAJB2a isoform (NP_001034639.1), causing the extension of the protein product by 83 amino acids (Fig. 2A). The variant sequence is also present deep in the 3′ UTR of the *DNAJB2b* transcript (NM_006736.6:c.1983 T > G), with no effects expected on the protein product. RNA sequencing (RNAseq) analysis of the proband’s muscle biopsy showed expression of the wild-type and mutant transcripts at the expected ratio (Fig. 2B), indicating that the mutant allele is normally expressed and does not affect RNA stability. RNAseq detected transcript variants 1 (*DNAJB2a*) and 2 (*DNAJB2b*) at a ~5:1 ratio, with no indication of altered isoform ratio or other *DNAJB2* splicing changes in the proband (Fig. 2B).

To exclude causative variants outside the MyoCap panel, whole-exome sequencing was performed for all three available family members (Supplementary Material, Fig. S1). Apart from the *DNAJB2* variant, the only other variant predicted as likely pathogenic and segregating with the disease was a heterozygous deletion abolishing the start codon of the *FIBP* gene. As recessive loss-of-function mutations in *FIBP* cause Thauvin–Robinet–Faivre syndrome, a congenital syndrome characterized by tall stature, intellectual disability and renal anomalies (22,23), the identified variant is unlikely relevant for the neuromyopathy phenotype in our family.

The *SGCE* variant NM_003919.3:c.1046_1047del:p.(Arg349Lysfs^*^29), predicted by VarSome as likely pathogenic, was identified by MyoCap and by exome sequencing in the proband but not in his brother or sister. Dominant *SGCE* mutations are a reported cause of myoclonus–dystonia, a phenotype not compatible with that seen in our patients (24). The identified frameshift variant affects an alternatively spliced exon and was according to RNAseq only present in ~25% of *SGCE* transcripts in the proband’s muscle biopsy.

### Western blotting and immunofluorescence analysis of patient biopsy

**Figure 3:**
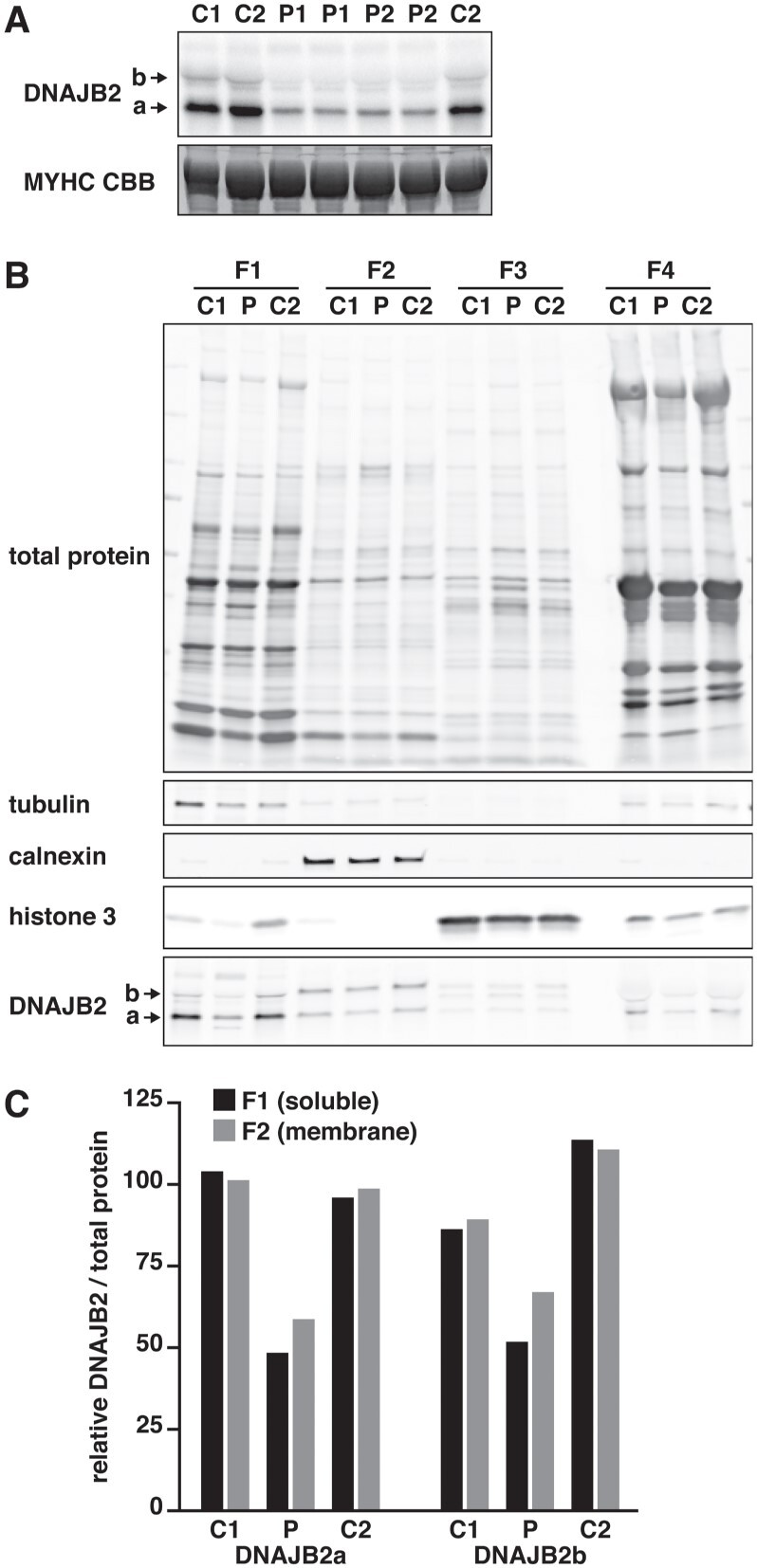
Western blotting of patient biopsy. (**A**) Western blotting of a muscle biopsy from the proband (P) revealed a reduced amount of both DNAJB2a and DNAJB2b proteins compared with pooled control (C), and no detectable mutant protein. P1 and P2 are independently prepared samples from the same biopsy. Post-blotting Coomassie staining of the myosin heavy chain (MYHC CBB) is shown as loading control. (**B**) Control (C1, C2) and proband (P) muscle biopsies were fractionated with the ProteoExtract Subcellular Proteome Extraction Kit to cytosolic (F1), membrane/organelle (F2), nuclear (F3) and cytoskeletal/insoluble (F4) fractions and analysed by western blotting. Total protein, tubulin, calnexin and histone 3 are shown as loading and fractionation controls. (**C**) The levels of DNAJB2 relative to total protein were quantified from the F1 and F2 fractions in (B) and represented normalized to the mean of control samples. Both DNAJB2 isoforms showed a ~50% reduction in the biopsy of the proband.

Western blotting was performed to evaluate the effects of the DNAJB2 mutation in a muscle biopsy from the proband. The extended protein product was not present at a detectable level in total muscle lysate. Interestingly, however, the protein levels of both DNAJB2a and DNAJB2b isoforms appeared reduced by >50% compared with pooled control muscle (Fig. 3A). Prompted by the membrane localization of mutant DNAJB2 (see below), we performed subcellular fractionation of patient and control muscles. Although the extended protein was not detectable in the membrane fraction, the results supported the decrease of both DNAJB2a and DNAJB2b in the soluble and membrane fractions (Fig. 3B and C).

In immunofluorescence microscopy (not shown), the biopsy of the proband showed myopathic features including fibre size variation and internal nuclei. Occasional rimmed-vacuolar fibres were encountered, however, without major SQSTM1/p62 or TARDBP accumulation pathology. Immunofluorescent analysis did not show significant DNAJB2 or DNAJB6 accumulation pathology.

### Characterization of the mutant protein

**Figure 4:**
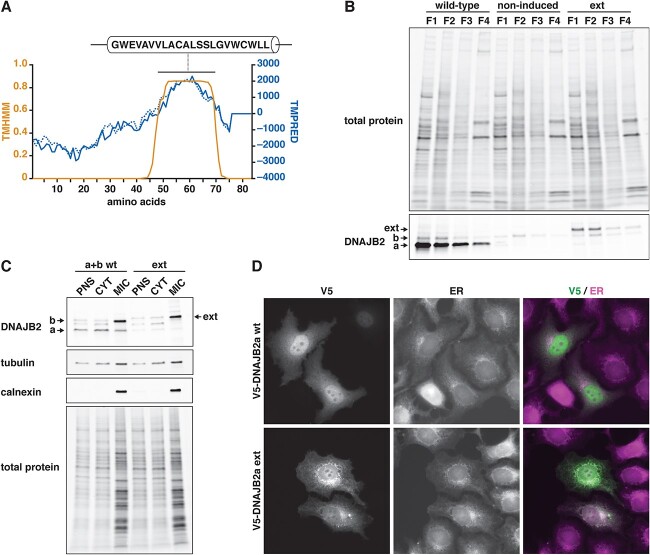
Membrane localization of mutant DNAJB2. (**A**) *In silico* prediction. The amino acid sequence of DNAJB2a p.^*^278Glyext^*^83 was analysed with transmembrane helix prediction algorithms. The graph shows the scores from TMHMM (orange trace) and TMPRED (blue solid trace, in–out orientation, dashed trace, out–in) for the 83 amino acid extension. (**B**) Stably transfected C2C12 myotubes induced to express wild-type or p.^*^278Glyext^*^83 (ext) DNAJB2a, and non-induced control cells, were fractionated with the ProteoExtract Subcellular Proteome Extraction Kit to cytosolic (F1), membrane/organelle (F2), nuclear (F3) and cytoskeletal/insoluble (F4) fractions. Endogenous and overexpressed DNAJB2a were predominantly cytosolic, whereas p.^*^278Glyext^*^83 was enriched in the membrane fraction similarly to endogenous DNAJB2b. (**C**) T-REx 293 cells were transfected with a combination of wild-type DNAJB2a and DNAJB2b (a + b wt) or DNAJB2a p.^*^278Glyext^*^83 (ext) and fractionated. Wild-type DNAJB2a was mostly found in the cytosolic (CYT) fraction, whereas DNAJB2b and p.^*^278Glyext^*^83 were enriched in the microsomal (MIC) fraction, similarly to the ER marker calnexin. PNS, post-nuclear supernatant. (**D**) In transfected HeLa cells, wild-type (wt) V5-DNAJB2a showed diffuse nuclear and cytoplasmic localization, whereas p.^*^278Glyext^*^83 (ext) partially colocalized with the endoplasmic reticulum (ER) visualized with the Cytopainter ER staining kit.

To understand the effects of the p.^*^278Glyext^*^83 extension, we analysed the properties of the mutant protein *in silico* and in cell culture experiments. Sequence analysis with the TMPRED and TMHMM algorithms predicted a transmembrane helix in the C-terminal extension (Fig. 4A). In line with this, subcellular fractionation of stably transfected C2C12 myotubes revealed increased localization of DNAJB2a p.^*^278Glyext^*^83 to the membrane fraction, whereas overexpressed and endogenous wild-type DNAJB2a were mostly found in the soluble cytosolic fraction (Fig. 4B). Similarly, the overexpressed p.^*^278Glyext^*^83 protein showed enrichment to the microsomal fraction in T-REx 293 cells (Fig. 4C) and partially colocalized with the ER in HeLa cells (Fig. 4D).

**Figure 5:**
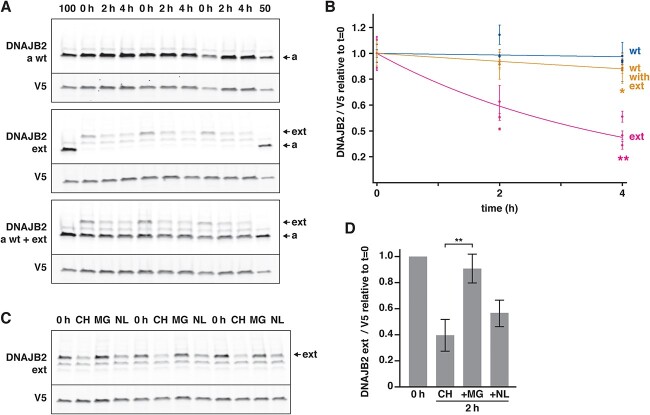
Turnover studies. (**A**–**B**) Wild-type (wt) or p.^*^278Glyext^*^83 (ext) DNAJB2a were expressed in T-REx 293 cells alone or in combination, and their levels were assayed at 0, 2 and 4 h of cycloheximide treatment. (A) A representative experiment performed in triplicate. 100 and 50 indicate a normalization sample at 100% and 50% loading, common for all three blots. (B) Quantification of three replicate experiments. The level of DNAJB2 was normalized to the transfection marker (GFP-V5) and represented relative to the initial level (*t* = 0). Each data point represents the mean ± SD of one experiment performed in triplicate. Asterisks indicate significant differences in remaining protein amount at *t* = 4 compared with wild-type (2-tailed *t*-test; ^*^P = 0.029, ^*^^*^P = 0.001). DNAJB2 p.^*^278Glyext^*^83 showed an increased turnover rate and also increased the turnover of the co-expressed wild-type DNAJB2a. (**C**–**D**) T-REx 293 cells expressing DNAJB2 p.^*^278Glyext^*^83 were treated with cycloheximide alone (CH) or in combination with the proteasome inhibitor MG132 (MG) or lysosomal inhibitors (NH_4_Cl/leupeptin; NL) for 2 h. (D) The level of DNAJB2 was normalized to the transfection marker (GFP-V5) and represented relative to the initial level (*t* = 0). The graph shows means ± SD from three replicate experiments, each performed in triplicate. MG132 efficiently blocked the turnover of mutant DNAJB2 (^*^^*^P = 0.006, 2-tailed *t*-test).

The absence of detectable p.^*^278Glyext^*^83 in patient muscle and the lower steady-state levels of the mutant constructs in transfection experiments suggested increased turnover of the mutant protein. In line with this, cycloheximide chase experiments indicated dramatically increased turnover rate of mutant DNAJB2a p.^*^278Glyext^*^83 (Fig. 5A and B). Moreover, DNAJB2a p.^*^278Glyext^*^83 slightly accelerated the turnover of cotransfected wild-type DNAJB2a. The turnover of the mutant protein was efficiently blocked with the proteasomal inhibitor MG132, whereas lysosomal inhibitors showed little effect (Fig. 5C and D). Altogether, these findings indicate that DNAJB2a p.^*^278Glyext^*^83 undergoes rapid degradation through the ubiquitin–proteasome pathway and may also destabilize the wild-type protein, consistently with the decreased amount of DNAJB2 in patient muscle.

**Figure 6:**
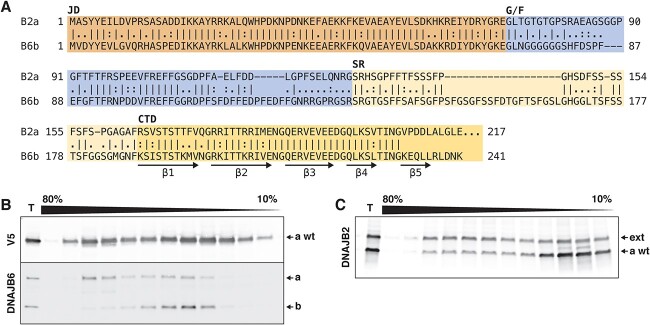
Oligomerization of DNAJB2. (**A**) Local sequence alignment of DNAJB2a (B2a) and DNAJB6b (B6b), with the coloured shadings indicating the J domain (JD), the glycine/phenylalanine-rich domain (G/F), the serine-rich region (SR) and the C-terminal domain (CTD) as defined for DNAJB6b by Karamanos *et al*. (25). Most of the region mediating DNAJB6b oligomerization (CTD β strands β1–β5) is highly similar between the two proteins. The C-terminal part of DNAJB2a (amino acids 218–277), not homologous to DNAJB6, is not shown. (**B**–**C**) Density gradient centrifugation of T-REx 293 cell lysates in 10–80% sucrose gradients. Fractionation profile of wild-type V5-DNAJB2a (a wt) suggests its oligomerization into polydisperse oligomers, similarly to endogenous DNAJB6 (a and b isoforms indicated) in the same samples (B). Co-expressed untagged wild-type (wt) and p.^*^278Glyext^*^83 (ext) DNAJB2a are both distributed throughout the gradient, albeit with somewhat different profiles (C). T, total samples.

A possible explanation for the dominant negative effect of DNAJB2a p.^*^278Glyext^*^83 could be co-oligomerization with the wild-type protein. Although oligomerization of DNAJB2 has not been reported, the related cochaperone DNAJB6 forms polydisperse oligomers through its CTD (25–27), which shows a high degree of similarity to DNAJB2 (Fig. 6A). Indeed, DNAJB2a constructs expressed in T-REx 293 cells showed polydispersity in sucrose density gradient centrifugation, supporting potential oligomerization of DNAJB2a (Fig. 6B). Although the fractionation profiles of wild-type and p.^*^278Glyext^*^83 DNAJB2a were somewhat dissimilar, both showed a polydisperse distribution in sucrose gradients, consistently with a possibility of co-oligomerization (Fig. 6C).

## Discussion

Our functional studies provide strong evidence for the abnormal behaviour of the DNAJB2a p.^*^278Glyext^*^83 protein, consistent with the idea that the identified *DNAJB2* variant is the molecular cause of dominantly inherited neuromyopathy in our family. The causative role of *DNAJB2* is further supported by the DNAJB2 protein deficiency observed in patient muscle. However, due to the small family size and unavailability of the parents’ DNA samples, contribution of other genetic factors to the phenotypic diversity in the family cannot be excluded.

Our proband presented with a combination of sensorimotor polyneuropathy and myopathy. The initial vitamin B_12_ deficiency may have aggravated the neurogenic component in the proband. However, considering the progressive course of the disease despite a timely and effective vitamin B_12_ supplementation, the absence of corresponding spinal cord changes in MRI and the presence of neuropathy also in the brother, the role of B_12_ deficiency is not major. The neuropathy symptoms seen in the proband are similar to those caused by recessive loss-of-function mutations in *DNAJB2*, albeit with later onset. The subjectively almost asymptomatic clinical phenotype in the proband’s brother defines the mild end of the spectrum, and it is likely that the walking difficulties in their mother were related to the same disease. In contrast to previous report of pyramidal tract signs in some dHMN patients with homozygous *DNAJB2* variants (16), our proband’s neurophysiological examinations point to central motor affection but without a clinical correlate. Again, vitamin B_12_ deficiency seems an unlikely cause of this in the absence of MRI abnormalities correlating to this measurement and with the deficiency corrected promptly.

The myopathological features and the prominent hyperCKemia in our proband suggest a concomitant myopathic process, not usually associated with DNAJB2 mutations, although elevated CK has been previously reported in individual patients with the c.14A > G p.(Tyr5Cys) (12) and c.184C > T p.(Arg62Trp) mutations (20). In the latter case, the patient showed neurogenic myopathology (angulated atrophy, fibre type grouping) with infrequent rimmed-vacuolar changes (20), suggesting a possible myopathic component. Additional studies will be needed to clarify the frequency and mechanisms of myopathy associated with DNAJB2 loss of function.

The recessive manifestation of *DNAJB2* null mutations suggests that ~50% reduction in DNAJB2 level is not alone sufficient to cause disease. Indeed, Saveri *et al*. showed reduced transcript and protein expression in lymphoblasts from a non-symptomatic heterozygous carrier of the c.145delG p.Val49Trpfs^*^25 variant (21). We therefore hypothesize that the DNAJB2 c.832 T > G p.(^*^278Glyext^*^83) mutation may cause disease through a combination of mechanisms. On one hand, the loss of functional DNAJB2a expression from the mutant allele combined with accelerated turnover of the wild-type protein via a dominant negative effect would lead to the DNAJB2 protein deficiency observed in our patient. On the other hand, the extended protein—notwithstanding its low steady-state level—could have an additional toxic gain-of-function effect. In a similar fashion, C-terminal extension mutations in HSPB8 cause disease through a gain-of-function mechanism even at an undetectable protein level (10,28,29).

Interestingly, dominant mutations in other JDP cochaperones, DNAJB6 (26,30,31) and DNAJB4 (32), also lead to muscle disease. These mutations likely disrupt the interaction between the G/F and J domains, leading to toxic gain-of-function by stalling the HSPA chaperone complex, and are associated with reduced turnover and/or accumulation of the mutant JDP (10,26,32–34). Given the accelerated turnover of mutant DNAJB2, the dominant toxicity in our case appears mechanistically distinct, and could possibly depend on mislocalized DNAJB2 interfering with PQC or calcium handling at the ER. Although our attempts to determine the membrane topology of the mutant protein on microsomal preparations remained inconclusive (not shown), the TMHMM prediction places the N-terminal major part on the protein ‘outside’, i.e. facing the ER lumen. That said, the mutant protein can be envisioned to exert a harmful effect in either orientation.

The apparent primary myopathic component and the relatively benign though progressive clinical course compared with DNAJB2 loss-of-function mutations likely reflect the fact that the identified variant is predicted to specifically affect DNAJB2a. We found this isoform, in line with previous work (6), to be the predominant one in skeletal muscle both at RNA and protein levels. In muscle, both the amount of the toxic protein and the dominant negative effect leading to loss of wild-type DNAJB2 would be more pronounced compared with neurons and other cell types where DNAJB2b is the predominant isoform.

In summary, the robust *in vitro* effects on protein localization and stability, and altered DNAJB2 level in patient muscle support classification of the *DNAJB2* variant NM_001039550.2:c.832 T > G p.(^*^278Glyext^*^83) as likely pathogenic. Although additional genetic or environmental factors may contribute to the disease phenotypes, the *DNAJB2* variant can be concluded to be the probable main cause of dominantly inherited neuromyopathy in our family.

## Materials and Methods

### Targeted high-throughput sequencing

Targeted high-throughput sequencing of genomic DNA was performed with the MyoCap panel (35) versions 5 (for the proband) and 6 (for his brother). The panels cover, respectively, 328 and 344 reported or candidate genes for myopathy (Supplementary Material, Table S2). Raw data were analysed by an in-house pipeline (35). VarSome (36) was used for variant interpretation.

### Whole-exome sequencing

Whole-exome sequencing was performed using the Twist Human Core Exome + RefSeq + Mitochondrial Panel (Twist Bioscience) at CeGaT (Tübingen, Germany) on Illumina NovaSeq 6000 (PE 2×100). Short variant (SNPs and indels) discovery was done on the Euformatics (Espoo, Finland) Orchestrator implementing the GATK Best Practices per sample pipeline. In brief, alignment of the raw reads was done using Burrows–Wheeler alignment tool on the University of California, Santa Cruz hg38 reference genome and variant calling was done using the Haplotype Caller. Variant annotation and automated classification were performed on the Euformatics omnomicsNGS system, which integrates data from multiple annotation sources and uses tools such as the Variant Effect Predictor (VEP v.107) for enriching every variant with annotations covering ~150 ‘dimensions’. Variant filtering was performed on omnomicsNGS (Supplementary Material, Fig. S1). VarSome (36) was used for variant interpretation.

### RNA sequencing

For RNAseq, total RNA was isolated from a gastrocnemius muscle biopsy of the proband with Qiagen RNeasy Plus Universal Mini Kit (Qiagen, Hilden, Germany). Total RNAseq library was prepared using the Illumina Ribo-Zero Plus rRNA Depletion kit (Illumina, Palo Alto, CA, USA) and run on a NovaSeq 6000 (Illumina) at the Oxford Genomics Centre, Wellcome Trust Institute, Oxford, UK, generating approximately 133 million 150 bp long reads (37). Sequences were mapped to Gencode.v39 human reference genome (based on ENSEMBL GRCh38.p13), using the STAR (v 2.7.0d) (38) two-pass method.

### Protein sequence analysis

The primary sequence of mutant DNAJB2 was analysed for transmembrane domains by TMPRED (http://embnet.vital-it.ch/software/TMPRED_form.html) and TMHMM (https://services.healthtech.dtu.dk/service.php?TMHMM-2.0). Local pairwise sequence alignment of DNAJB2a and DNAJB6b was performed with EMBOSS Matcher (https://www.ebi.ac.uk/Tools/psa/emboss_matcher/) using default parameters.

### SDS-PAGE and western blotting

For western blotting analyses, protein samples were separated in TGX minigels (Bio-Rad, Hercules, CA, USA) and transferred on nitrocellulose membranes with the Trans-Blot Turbo system (Bio-Rad). Total protein was stained with the Revert 700 Total Protein Stain (Li-Cor Biosciences, Lincoln, NE, USA) and scanned with the Odyssey scanner (Li-Cor). For muscle lysates, gels were stained post-blotting with Coomassie blue. Blots were stained with the following primary antibodies: calnexin Rb mAb C5C9 (Cell Signaling Technology, 2679, RRID:AB_2228381); DNAJB2 Rb pAb (Proteintech, 10 838-1-AP, RRID:AB_2277491); DNAJB6 Rb mAb [EPR17122] (Abcam, ab198995, RRID:AB_2924896); histone 3 Rb pAb (Abcam, ab1791, RRID:AB_302613); tubulin Rt mAb YL1/2 (Abcam, ab6160, RRID:AB_305328); V5 Ms mAb (Thermo Fisher, R960-25, RRID:AB_2556564); V5 Rb mAb D3H8Q (Cell Signaling Technology, 13202, RRID:AB_2687461). Detection was performed with fluorescent secondary antibodies using an Odyssey scanner or with HRP-conjugated secondary antibodies visualized by enhanced chemiluminescence. Image analysis was done in Fiji (39).

### Plasmid constructs

Tetracycline-inducible untagged and N-terminally V5-tagged DNAJB2a and DNAJB2b constructs in pCDNA5/FRT/TO (40) were a kind gift from Harm Kampinga. The sequence encoding the C-terminal extension was synthesized at Eurofins Genomics (Ebersberg, Germany) and added to the DNAJB2a constructs to create the p.^*^278Glyext^*^83 constructs. The inserts were transferred to pSBtet-Hyg (41) (a kind gift from Eric Kowarz; Addgene plasmid # 60508; RRID: Addgene_60 508) to create pSBtet-Hyg-DNAJB2a wild-type and p.^*^278Glyext^*^83. pEGFP-V5, encoding C-terminally V5-tagged green fluorescent protein, was constructed in pEGFP-C1.

### Histological analyses

The proband underwent gastrocnemius muscle biopsy at the age of 66 years. Conventional muscle biopsy procedure and histological analyses were performed, including haematoxylin/eosin, NADH diaforase and ATPase pH 4.6 stainings.

For immunofluorescence microscopy, cryosections prepared from the snap-frozen muscle biopsy were fixed using 4% paraformaldehyde (PFA) in phosphate buffered saline and were then immunostained. Primary antibodies were incubated overnight at +8°C and secondary antibodies 1 h at room temperature. Primary antibodies: DNAJB2 Rb pAb (Proteintech, 10838-1-AP, RRID:AB_2277491); DNAJB6 Rb mAb [EPR17122] (Abcam, ab198995, RRID:AB_2924896); TDP-43 Ms mAb (Sigma-Aldrich, WH00234, clone 2E2-D3, RRID:AB_1843869); p62 Rb pAb (Sigma-Aldrich, P0067, RRID:AB_1841064). Secondary antibodies: Alexa Fluor-488 and -546 conjugated secondary antirabbit and antimouse antibodies.

### Fractionation and western blotting of patient biopsy

For a total protein lysate, biopsied gastrocnemius muscle from the proband was homogenized in sample buffer (50 mm Tris–HCl pH 6.8; 4% sodium dodecyl sulphate, 8.7% glycerol, 10% 2-mercaptoethanol, bromophenol blue). DNAJB2 was analysed by western blotting, using post-blotting myosin heavy chain as loading control. For subcellular fractions, the biopsy was fractionated with the ProteoExtract Subcellular Proteome Extraction kit (Merck Millipore) as per the kit instructions. Tubulin, calnexin and histone 3 were used as markers for soluble, membrane and nuclear fractions, respectively.

### Immunofluorescence microscopy of HeLa cells

For comparing the localization of wild-type and mutant DNAJB2, HeLa cells seeded on coverslips were transfected with V5-tagged or untagged pCDNA5/FRT/TO–DNAJB2 constructs and fixed with 4% PFA the next day. The cells were stained with V5 or DNAJB2 antibodies, and counterstained with the Cytopainter ER Staining Kit—Red Fluorescence (ab139482; Abcam plc, Cambridge, UK). Images were acquired with Zeiss Axio Imager M2 using a 40× NA 1.30 objective.

### Microsome isolation

For studying the subcellular localization of the proteins, T-REx 293 cells were transfected with untagged pCDNA5/FRT/TO–DNAJB2 constructs (DNAJB2a p.^*^278Glyext^*^83 or a 1:1 mix of wild-type DNAJB2a and DNAJB2b). After ~20 h of tetracycline induction (500 ng/ml), cells were fractionated by ultracentrifugation essentially as described (42) and analysed by western blotting.

### C2C12 myotubes

To create C2C12 cells with inducible expression of wild-type or p.^*^278Glyext^*^83 mutant DNAJB2a, C2C12 myoblasts were cotransfected with pSBtet-Hyg-DNAJB2 and pCMV(CAT)T7-SB100 (43) (a kind gift from Zsuzsanna Izsvák; Addgene plasmid #34879, RRID: Addgene_34 879). Polyclonal pools of stably transfected cells were selected in growth medium [pyruvate-free Dulbecco’s modified Eagle medium (DMEM) with 20% fetal calf serum, Glutamax and penicillin/streptomycin] with 200 μg/ml hygromycin B and maintained in growth medium with 50 μg/ml hygromycin B. The cells were grown on collagen-coated plates to confluency and differentiated to myotubes in differentiation medium (pyruvate-free DMEM with 2% heat-inactivated horse serum, l-glutamine, penicillin/streptomycin and 10% Opti-MEM I) for 4 days. One day before harvest, the cells were induced with doxycycline (2 μg/ml) to express DNAJB2 or left uninduced. The cells were fractionated with the ProteoExtract Subcellular Proteome Extraction kit according to the kit instructions and analysed by western blotting.

### Protein turnover assays

For turnover studies, T-REx 293 cells were cotransfected with pCDNA5/FRT/TO–DNAJB2 construct(s) and pEGFP-V5. After 16 h of tetracycline induction (200 ng/ml), the medium was replaced with a chase medium containing 40 μg/ml cycloheximide (CHX). Immediately (*t* = 0) and at different time points (2, 4 and 6 h) of CHX treatment, cells were rinsed and frozen at −80°C. To block proteasomal or lysosomal protein turnover pathways, 10 μm MG132 or 20 mm NH_4_Cl + 100 μm leupeptin was included in the chase medium. DNAJB2 and GFP-V5 were analysed from the cell lysates by fluorescent western blotting using an Odyssey scanner. The amount of DNAJB2 was normalized to GFP-V5 (which does not turn over significantly during the chase period) to correct for transfection efficiency and was expressed relative to *t* = 0.

### Sucrose density gradient centrifugation

To study DNAJB2 oligomerization, DNAJB2 constructs were expressed in T-REx 293 cells for 2 days and analysed by density gradient ultracentrifugation in 10–80% sucrose gradients as described previously for DNAJB6 (26).

## Supplementary Material

HMG-2023-CE-00031_rev290323_Suppl_fig1_ddad058

HMG-2023-CE-00031_rev290323_Suppl_table1_ddad058

HMG-2023-CE-00031_rev290323_Suppl_tables2-3_ddad058

## Data Availability

The data are available upon reasonable request. The DNAJB2 c.832 T > G p.(^*^278Glyext^*^83) variant has been submitted to LOVD by individual ID 00430268.
