# Supplementary material for: Extension of the DNAJB2a isoform in a dominant neuromyopathy family
Source: Hum Mol Genet. 2023 Apr 18;32(21):3029–39. doi: 10.1093/hmg/ddad058 (PMC10586202; doi:10.1093/hmg/ddad058)
Supplement: HMG-2023-CE-00031_rev290323_Suppl_fig1_ddad058 [file hmg-2023-ce-00031_rev290323_suppl_fig1_ddad058.pdf]

Supplementary figure 1: Sequential filtering steps for the analysis of exome sequencing data

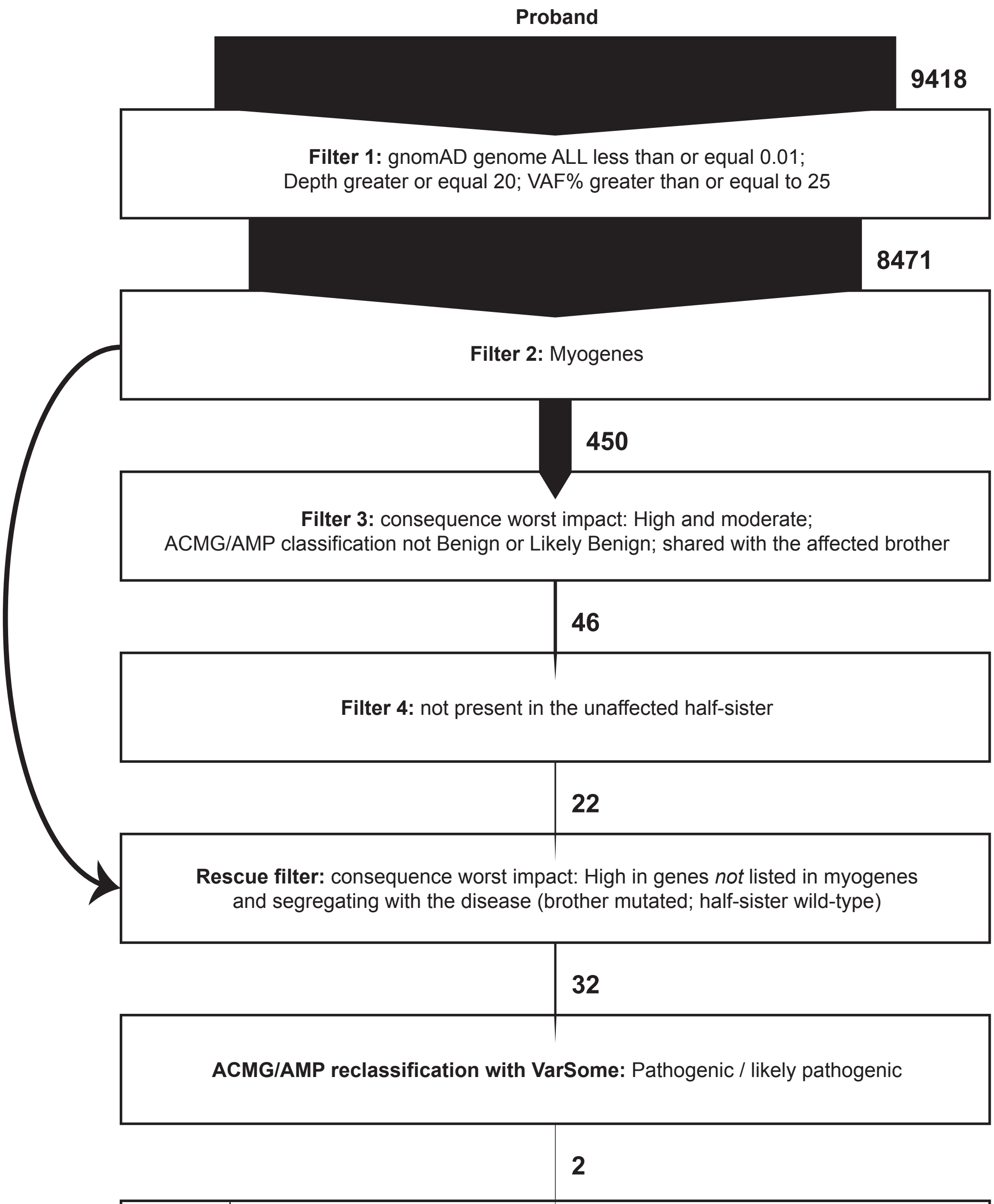

gene	variant
FIBP	NM_004214.5:c.-29_8del
DNAJB2	NM_001039550.2:c.832T>G(p.Ter278Glyext*83)

Overview of the variant filtering strategy showing the number of variants in each filtering step:

Filter 1 includes frequency, depth and variant allele frequency (VAF).

Filter 2 focuses on a virtual gene panel “Myogenes” including 1891 causative or candidate genes (all reported neuromuscular disease genes plus genes highly expressed in muscle; gene list in Supplementary table 3).

Filter 3 considers the variant impact (High and moderate as a worst impact), AMCG/AMP-based classification (as provided by the omnomicsNGS system) and the segregation in the affected proband´s brother.

Filter 4 considers segregation data in the proband´s unaffected half-sister.

Rescue filter: Variants having a disruptive impact on the protein (High consequence worst impact), segregating with the disease, are rescued and re-analyzed even if they are not located in genes listed in the virtual gene panel.

After re-classification with Varsome, only two variants are classified as pathogenic/likely pathogenic.
