# Supplementary material for: Extension of the DNAJB2a isoform in a dominant neuromyopathy family
Source: Hum Mol Genet. 2023 Apr 18;32(21):3029–39. doi: 10.1093/hmg/ddad058 (PMC10586202; doi:10.1093/hmg/ddad058)
Supplement: HMG-2023-CE-00031_rev290323_Suppl_table1_ddad058 [file hmg-2023-ce-00031_rev290323_suppl_table1_ddad058.pdf]

Sarparanta et al. Extension of the DNAJB2a isoform in a dominant neuromyopathy family

Supplementary table 1: Neurophysiological findings

Exam	First visit	A month later	31 months later	43 months later
**Neurography**				
R median	M: normal, S: SNAP↓		M: DML↑, S: no SNAP	M: DML↑; FWL↑; S: SNAP↓
R peroneal	M: normal		M: normal	M: normal
L tibial	M: CMAP↓ (pCB), FWL↑		M: CMAP↓ (pCB), FWL↑	M: CMAP↓, FWL↑
R ulnar	S: no SNAP	M: DML↑, FWL↑, CMAP↓(Erb)	S: no SNAP	FWL↑
L ulnar		M: DML↑, FWL↑, CMAP↓(Erb), mNCV↓ (axillary)	FWL↑	FWL↑
R sural	S: no SNAP		S: no SNAP	S: no SNAP
L sural	S: no SNAP			S: no SNAP
**Electromyography**				
R deltoid	MUP duration↑	MUP duration↑		
L deltoid			MUP duration↑	MUP duration↑, amplitude↑
R vastus lateralis			MUP duration↑, amplitude↑, PSA	MUP amplitude↑, PSA
L vastus lateralis	normal	normal		
L extensor digitorum communis		MUP duration↑	MUP duration↑, amplitude↑	MUP duration↑
R tibialis anterior		normal		Normal
L tibialis anterior			MUP duration↑, PSA	
R Paravertebal Th12		No PSA	No PSA	No PSA
L Paravertebal Th12		No PSA	No PSA	No PSA
**Transcranial magnetic stimulation**				
R abductor digitorum V		TCT↑, CCT↑	TCT: normal, CCT normal	TCT↑, CCT↑
L abductor digitorum V		TCT↑, CCT↑	TCT↑, CCT:normal	TCT↑, CCT↑
R tibialis anterior		TCT↑, CCT↑	No potential	No potential
L tibialis anterior		TCT↑, CCT↑	TCT↑, CCT:normal	No potential

CCT: central conduction time; CMAP: compound muscle action potential; DML: distal motor latency; FWL: F wave latency; L: left; M: motor; mNCV: motor nerve conduction velocity; MUP: motor unit potential; pCB: partial conduction block; PSA: pathologic spontaneous activity; R: right; S: senory; SNAP: sensory nerve action potential; TCT: total conduction time;
